# Emerging roles of plant-derived extracellular vesicles in the management of dental diseases

**DOI:** 10.20517/evcna.2026.145

**Published:** 2026-09-28

**Authors:** Qingping Xi, Siyuan Wang, Qiongling Shi, Hui Wang, Ping Sun, Yan Jin, Yang Ma

**Affiliations:** ^1^Stomatology Hospital, School of Stomatology, Zhejiang University School of Medicine, Zhejiang Provincial Clinical Research Center for Oral Diseases, Zhejiang Key Laboratory of Oral Biomedical, Hangzhou 310016, Zhejiang, China.; ^2^Applied Oral Sciences & Community Dental Care, Faculty of Dentistry, The University of Hong Kong, Hong Kong, China.; ^3^State Key Laboratory of Oral & Maxillofacial Reconstruction and Regeneration, National Clinical Research Center for Oral Diseases, Shaanxi Key Laboratory of Stomatology, Center for Tissue Engineering, School of Stomatology, The Fourth Military Medical University, Xi’an 710032, Shaanxi, China.; ^#^These authors contributed equally to this work.

**Keywords:** Plant-derived extracellular vesicles, dental diseases, microorganisms, inflammation, tissue regeneration

## Abstract

Dental diseases, including dental caries, pulpitis, and periodontitis, can cause pain and tooth loss, compromising mastication, speech, aesthetics, and quality of life. These diseases have also been associated with systemic disorders, including Alzheimer’s disease. Conventional interventions, such as restorative procedures, root canal treatment, and periodontal scaling, have inherent limitations, underscoring the clinical need for novel therapeutic strategies. Plant-derived extracellular vesicles (PDEVs) have recently attracted considerable attention owing to their wide availability, cost-effectiveness, favourable biosafety profile, and capacity to mediate cross-kingdom communication. PDEVs exert diverse biological effects, including antimicrobial, anti-inflammatory, immunomodulatory, and tissue-regenerative activities. For example, ginger-derived PDEVs suppress the growth and virulence of *Porphyromonas gingivalis* through their lipid and miRNA cargoes, thereby alleviating periodontitis in mice. Given that effective dental therapy requires antimicrobial action, anti-inflammatory effects, immunomodulation, and tissue regeneration, PDEVs are promising therapeutic modalities. This review summarises the isolation, characterisation, composition, and biological functions of PDEVs, and discusses their potential applications in the management of dental diseases.

## INTRODUCTION

A 2022 World Health Organization (WHO) report estimates that approximately 3.5 billion people are affected by dental diseases, marking a major global health challenge^[1]^. Oral diseases, such as dental caries, pulpitis, and periodontitis, not only compromise functions, including mastication, speech, and aesthetics, but also impair social interaction and psychological well-being. Furthermore, oral pathogens can induce or exacerbate systemic conditions, including cardiovascular disease, Alzheimer’s disease, and diabetes, thereby endangering overall health and compromising quality of life^[2,3]^. In recent decades, therapeutic strategies utilizing natural foods and plant-based products have garnered substantial attention. This trend is exemplified by extensive research dedicated to fresh herbs and botanical derivatives. Notably, the WHO reports that at least 25% of all modern medicines are derived, either directly or indirectly, from medicinal plants^[4]^, underscoring the pivotal role of botanical sources in modern medicine. Recently, plant-derived extracellular vesicles (PDEVs) have emerged not only as potential therapeutic biomaterials for various diseases such as cancer and inflammatory bowel disease, but also as drug carriers^[5]^. Evidence indicates that PDEVs can be internalised by mammalian cells, subsequently modulating host cellular processes^[6]^. However, research regarding the applications of PDEVs in dental medicine remains nascent. This review details the methods currently employed to isolate and characterise PDEVs and summarises the current knowledge of their biological functions. We further discuss the development of PDEVs as therapeutic agents and their potential applications in the management of dental diseases.

## LANDSCAPE OF PDEVs

Terms such as PDEVs, plant-derived nanovesicles (PDNVs), plant-derived exosome-like nanovesicles (PELNs), and plant-derived vesicles (PDVs) have been used inconsistently to describe nano- and microsized vesicular particles obtained from plant materials. Herein, PDEVs are utilised as a pragmatic umbrella term to align with existing published literature. It should be emphasised that the commonly cited 50-1,000 nm size range is purely descriptive and does not correspond to a biologically homogeneous vesicle population. Since exosomes and microvesicles are classified primarily according to their biogenetic routes rather than particle dimensions, vesicles with uncharacterised origins should be categorised as small (< 200 nm) or medium/large (> 200 nm), consistent with the Minimal Information for Studies of Extracellular Vesicles (MISEV) recommendations^[7,8]^.

The earliest record of PDEVs dates back to 1967, when Halperin *et al*. visualised extracellular vesicular structures in carrot cells^[9]^. In 2009, Regente *et al*. identified exosome-like membrane-bound vesicles in sunflower apoplastic fluid, which were postulated to mediate intercellular communication^[10]^. Following these seminal findings, PDEVs have become a rapidly expanding research focus. Studies in *Arabidopsis thaliana* have identified distinct extracellular vesicle (EV) subpopulations, including TET8-positive and PEN1-positive vesicles, as well as EXPO-derived vesicles associated with EXO70E2, suggesting different biogenetic pathways and cargo profiles^[11-13]^. However, these markers have not been validated across plant species, and conventional tissue-disruption methods generally yield heterogeneous vesicle populations of uncertain biological origin. Although vesicle size may influence cargo composition, cellular uptake, biodistribution, and clearance^[14,15]^, direct comparisons of size-fractionated PDEVs remain limited^[16]^. Therefore, no diameter range can currently be considered universally optimal for therapeutic use. Nevertheless, defining and reporting specified size ranges may improve batch consistency, dose standardisation, and reproducibility^[7,16]^. Further studies comparing size-defined fractions from the same plant source are required to establish application-specific size criteria.

## PREPARATION AND ISOLATION OF PDEVs

### Preparation of PDEVs

A diverse array of plant materials can be utilised, including roots, stems, leaves, flowers, bark, fruits, and seeds, in fresh or processed forms^[17]^. Three principal techniques are widely used to process plant tissues and isolate PDEVs^[18]^. The first method involves mechanical disruption, such as blending, grinding, or pressing, to obtain crude plant extracts. Following extraction, the homogenate undergoes initial low-speed centrifugation to eliminate large fibres and cellular debris. The clarified supernatant is subsequently collected and subjected to ultracentrifugation (UC) to isolate the PDEVs. This approach offers operational simplicity, high efficiency, and high yield. However, the mechanical disruption of the cell wall often induces simultaneous rupture of the plasma membrane. This results in contamination with intracellular components and compromised vesicular membrane integrity^[5,19]^. The second strategy relies on enzymatic degradation of the plant cell wall, either alone or combined with mild mechanical processing, to liberate EVs trapped within the cell-wall matrix. Plant tissues are cut into small pieces and incubated with cell-wall-degrading enzymes under gentle agitation. The digested mixture is then filtered and subjected to low- and medium-speed centrifugation to remove tissue debris, yielding a clarified PDEV-containing supernatant for subsequent purification. Zhao *et al*. treated *Morinda officinalis* root tissues with cellulase, pectinase and hemicellulase, and achieved approximately three-fold higher particle yields per gram of tissue relative to conventional grinding, along with a smaller and more homogeneous size distribution^[20]^. More recently, the Phyto-EVpure enzymatic pulping method has enhanced EV yield and purity compared with grinding and has been validated across 17 medicinal plant species, diverse tissue types, and fresh, frozen, and dried starting materials^[21]^. This strategy is particularly beneficial for fibrous, desiccated, scarce or high-value plant materials. Nevertheless, enzyme type and concentration, digestion duration, pH and osmotic conditions need to be optimised for each plant species and tissue type. Over-digestion can disrupt the plasma membrane and trigger leakage of intracellular components, whereas residual enzymes may interfere with downstream analytical assays. Accordingly, residual enzyme removal and rigorous quality controls are indispensable. The third method utilises apoplastic washing fluid (AWF). In this protocol, refined by Rutter *et al*. for *Arabidopsis*, leaves are vacuum-infiltrated with an isolation buffer, surface-dried, and subjected to low-speed centrifugation to collect AWF for EV purification^[22]^. Although this infiltration method yields vesicles with preserved structural integrity and minimal cytoplasmic contamination, it is limited by its operational complexity, extended processing time, and low yield. In summary, extraction protocols must be tailored to plant morphology. Succulent fruits (e.g., grapefruit^[23]^) can be directly pressed, whereas fibrous, low-moisture tissues like roots and stems (e.g., garlic^[19]^) require grinding with phosphate-buffered saline to facilitate extraction. For leaves (e.g., *Arabidopsis thaliana*^[22]^), apoplastic fluid is collected via vacuum infiltration-centrifugation.

### Isolation of PDEVs

Diverse methodologies are employed for the isolation and purification of plant-derived EVs, with UC being the most prevalent and well-documented technique. Additional techniques include gradient ultracentrifugation (GUC), Polyethylene glycol (PEG)-based precipitation, ultrafiltration (UF), size-exclusion chromatography (SEC), and immunoaffinity capture [Figure 1].

**Figure 1:**
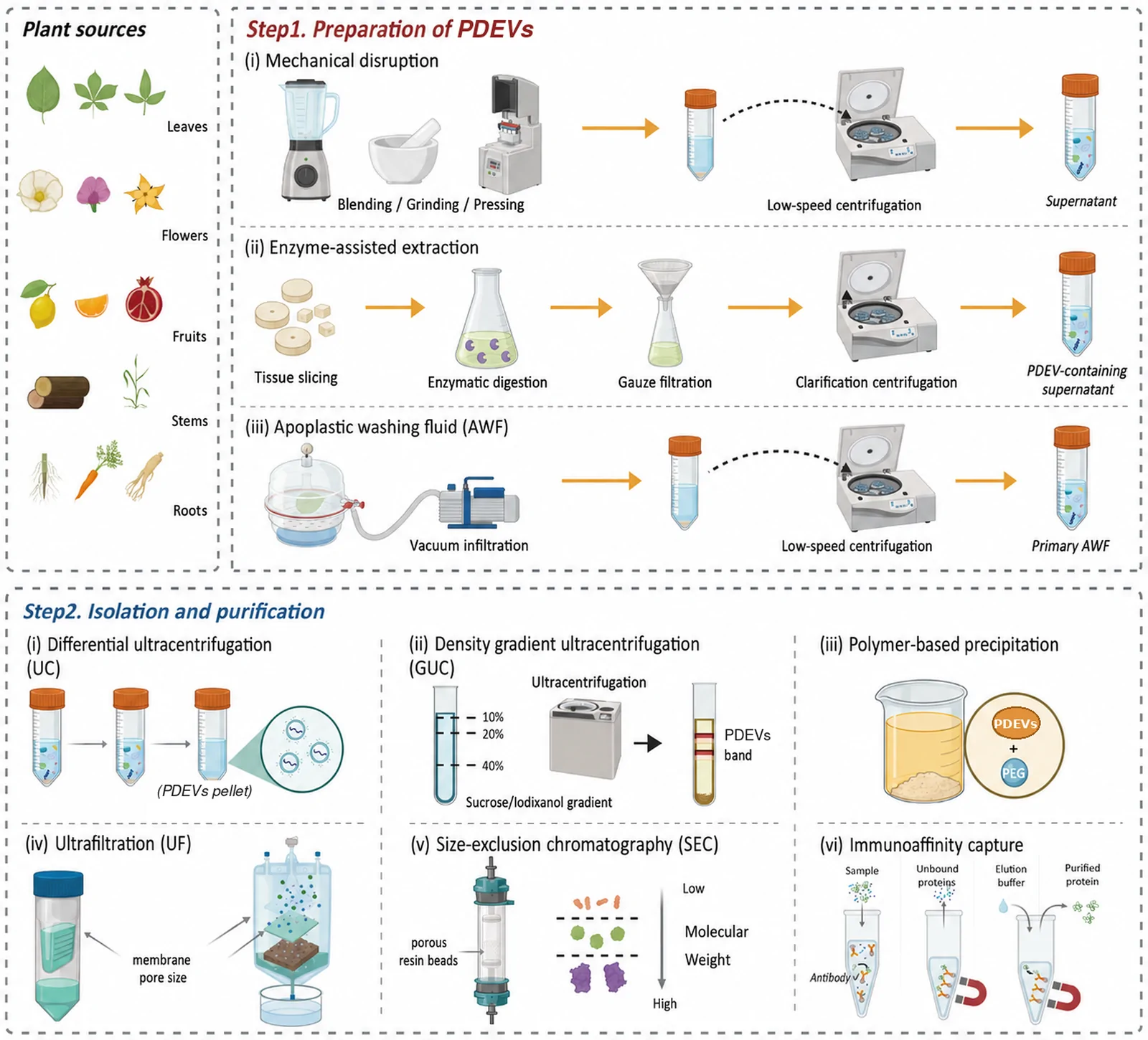
Preparation, isolation, and purification of PDEVs. Step 1 illustrates: (i) mechanical disruption followed by low-speed centrifugation; (ii) enzyme-assisted extraction involving tissue slicing, enzymatic digestion, filtration, and clarification centrifugation; and (iii) AWF collection by vacuum infiltration-centrifugation. Step 2 shows: (i) differential UC; (ii) density-GUC; (iii) polymer-based precipitation; (iv) UF; (v) SEC; and (vi) immunoaffinity capture. Created with BioRender.com and subsequently modified and finalised using Adobe Illustrator. https://BioRender.com/bygh8tl. AWF: Apoplastic washing fluid; GUC: gradient ultracentrifugation; PDEVs: plant-derived extracellular vesicles; SEC: size-exclusion chromatography; UC: ultracentrifugation; UF: ultrafiltration.

#### UC

Differential UC remains the "gold standard" for isolating PDEVs. This method fractionates vesicle subtypes by applying sequentially increasing centrifugal forces. A typical protocol initiates with low-speed centrifugation to eliminate cell debris, followed by medium-speed steps to pellet larger organelles. Finally, ultrahigh-speed centrifugation (typically ≥ 100,000 × *g*) pellets the nanosized PDEVs. A key advantage of this approach is the absence of exogenous reagents, which facilitates isolating vesicles in their native state. The protocol is also technically straightforward. However, it necessitates specialised instrumentation, is labour-intensive, and may compromise vesicle integrity due to high shear forces. Furthermore, co-sedimentation of non-vesicular contaminants often results in samples of suboptimal purity^[24]^.

#### GUC

Density gradient centrifugation fractionates PDEVs based on buoyant density, yielding high-purity samples from crude extracts. Samples are loaded onto a pre-formed gradient (e.g., sucrose or iodixanol) and centrifuged, causing PDEVs to band at their specific buoyant densities for collection. Although this method preserves vesicle integrity and purity, it is technically complex, low-yielding, and cost-prohibitive. Thus, it is frequently combined with differential centrifugation to optimise the balance between efficiency and purity^[25,26]^.

#### Polymer-based precipitation

Polymer-based precipitation isolates EVs by altering their solubility and dispersibility using exclusion agents, with PEG being the most prevalent polymer. These agents entrap vesicles within a mesh-like network, reducing their solubility and facilitating retrieval via low-speed centrifugation^[25]^. This method yields PDEVs with characteristics comparable to those obtained via differential UC, and yield can be substantially improved through protocol optimisation, such as pH adjustment^[27,28]^. Although polymer-based precipitation is simpler, more rapid, and more cost-effective than UC for large-volume samples, it generally results in lower purity^[29]^.

#### UF

UF is a pressure-driven technique that separates PDEVs based on size and molecular weight using microporous membranes with defined pore cutoffs. In this process, centrifugal force or applied pressure drives the sample through UF membranes, selectively retaining or passing vesicles based on size. This method enables efficient purification, concentration, and fractionation of PDEVs from crude samples based on hydrodynamic diameter^[30]^. UF offers a simple, rapid, and cost-effective alternative to UC, preserving PDEVs’ integrity and function without necessitating specialised instrumentation. However, size-based selectivity leads to the co-isolation of protein aggregates and particles of similar dimensions, thereby limiting final purity. Practical challenges include membrane clogging by concentrated or viscous samples, rendering the method most suitable for pre-clarified or dilute solutions^[8,30,31]^.

#### SEC

SEC fractionates particles based on hydrodynamic diameter. This method isolates EVs by exploiting the differential passage of particles through the porous resin beads of a chromatography column^[32]^. Owing to its simplicity, efficiency, ability to yield high-purity samples, and preservation of EV structure and function, SEC has emerged as a promising complementary technique for purifying PDEVs^[33]^. SEC can be combined with ultrafiltration, UC, or polymer-based precipitation and is widely applied for purifying vesicles from diverse plant species^[34,35]^.

#### Immunoaffinity capture

Immunoaffinity capture isolates and enriches EVs utilising antibody-conjugated beads targeting surface-specific protein markers. Although widely used in mammalian research because characteristic proteins, lipids, or polysaccharides are well characterised, this approach enables high-specificity EV subtyping. For example, following the identification of TET8 - a tetraspanin structurally homologous to mammalian CD63 - in *Arabidopsis*, TET8-positive vesicles were successfully isolated using magnetic beads coated with antibodies targeting the extracellular EC2 domain of the protein. Analysis of the captured vesicles revealed an enrichment of small RNAs and RNA-binding proteins^[11]^. Although immunoaffinity capture offers high specificity, its application in plant studies is hindered by the scarcity of antibodies targeting plant-specific markers.

#### Other methods

Yang *et al*. developed an electrophoretic-dialysis method for isolating PDEVs, utilising a 300 kDa cutoff dialysis bag combined with an electric field^[36]^. This approach enabled the extraction of lemon-derived extracellular vesicles directly from lemon juice, offering a rapid and instrument-efficient alternative to conventional isolation techniques. Additionally, a hydrophobic interaction chromatography method employing capillary-channelled polymer fibre tips facilitates the rapid isolation of PDEVs. This solid-phase extraction method, performed with a benchtop centrifuge, yields over 1 × 10^10^ particles from a 100 μL sample. The protocol successfully isolated PDEVs with an average diameter of 189 nm from 20 common fruits and vegetables^[37]^.

### Comparative evaluations and method selection

Direct comparisons of PDEV isolation methods remain limited, and no single approach is universally superior. In a four-method comparison of ginger-derived nanovesicles, UC yielded the highest protein-based recovery and favourable stability, whereas membrane filtration achieved the highest particle concentration. UC-isolated vesicles also displayed the most potent *in vitro* antiproliferative activity against lung cancer cells^[38]^. A comparison between UC and PEG precipitation using tobacco-derived vesicles revealed broadly comparable particle sizes and concentrations. PEG precipitation mitigated vesicle aggregation yet increased the risk of co-isolating soluble proteins^[39]^. Similarly, PEG6000 precipitation recovered approximately 60%-90% of the material obtained via UC from ginger, generating nanoparticles with comparable physicochemical properties and biological activity; nevertheless, residual PEG was still detectable following dialysis^[27]^.

Method selection should therefore be guided by the intended application. UC remains a well-established strategy for laboratory-scale and mechanistic studies, whereas PEG precipitation offers a simpler and potentially scalable alternative when recovery and throughput are prioritised. For applications requiring higher purity, additional density-gradient purification may be advantageous. For instance, sucrose- or iodixanol-based density-gradient UC efficiently separated tomato-derived nanovesicles from co-isolated viral particles^[40]^. Further standardised head-to-head comparisons across diverse plant sources are needed to evaluate particle recovery, purity, cargo preservation, biological activity and scalability.

## CHARACTERISATION OF PDEVs

PDEVs typically exhibit cup-shaped, spherical, or ovoid morphologies. These structures were visualised using scanning electron microscopy (SEM), transmission electron microscopy (TEM), cryo-electron microscopy (cryo-EM), and atomic force microscopy (AFM). SEM facilitates the observation of surface topography, whereas TEM is employed to analyse internal ultrastructure. However, the dehydration steps required for TEM sample preparation can induce shrinkage and morphological distortion. Nevertheless, TEM remains indispensable for high-resolution nanoscale imaging. Cryo-EM, which analyses EV morphology at cryogenic temperatures, circumvents dehydration-induced damage, thereby preserving native structural integrity. AFM offers high-resolution analysis of individual PDEVs, enabling 3D topographical visualisation and the measurement of nanomechanical properties such as adhesion and stiffness. The size distribution of PDEVs is frequently assessed using complementary techniques such as dynamic light scattering (DLS), nanoparticle tracking analysis (NTA), and laser transmission spectroscopy (LTS). DLS determines the average hydrodynamic diameter by analysing intensity fluctuations resulting from Brownian motion. In contrast, NTA visualises and quantifies individual EVs, providing direct insights into vesicle concentration and size distribution^[29]^. Most experimentally characterised PDEVs exhibit particle sizes of approximately 30-500 nm^[16,41]^. This represents a commonly observed distribution rather than a strict boundary, whereas the broader 50-1,000 nm range in Section “LANDSCAPE OF PDEVs” encompasses less common larger vesicles. Measured size may also vary with the plant source, isolation procedure, and analytical technique^[7,42]^. Zeta-potential measurement is a well-established technique for characterising PDEVs, as it enables the assessment of their surface charge and colloidal stability^[43,44]^. The magnitude of the zeta potential indicates vesicle aggregation propensity in suspension, facilitating the evaluation of formulation and storage stability^[43]^. Nevertheless, zeta potential is highly sensitive to experimental variables such as pH, ionic strength, buffer composition and particle concentration, necessitating careful control and full reporting of these experimental parameters^[43,44]^.

Surface marker proteins are pivotal for identifying EVs. While tetraspanins such as CD63, CD81, and CD9, alongside TSG101, are well-established biomarkers for mammalian EVs, PDEVs exhibit a less defined and more heterogeneous protein profile. Proteomic studies have identified hydrolytic and membrane-transport proteins in PDEVs, together with plant-specific proteins involved in cell-wall remodelling. Chloroplast-associated proteins have also been detected in some preparations, although their presence may partly reflect intracellular contamination or stress-related cargo^[45,46]^. Given its structural homology to mammalian CD63 and its enrichment in plant EVs, TET8 is considered a putative plant EV biomarker^[47]^. Pinedo *et al*. suggested that common plant protein families, including heat shock protein 70 (HSP70), GAPDH, and S-adenosyl-homocysteinase, may serve as surface markers for plant EVs^[8]^. Although diverse protein profiles have been identified in PDEVs, establishing specific and universal protein markers remains a substantial challenge.

## COMPOSITION OF PDEVs

Four major components have been identified in PDEVs: lipids, proteins, nucleic acids, and metabolites. This section discusses their roles in vesicle function and stability [Figure 2].

**Figure 2:**
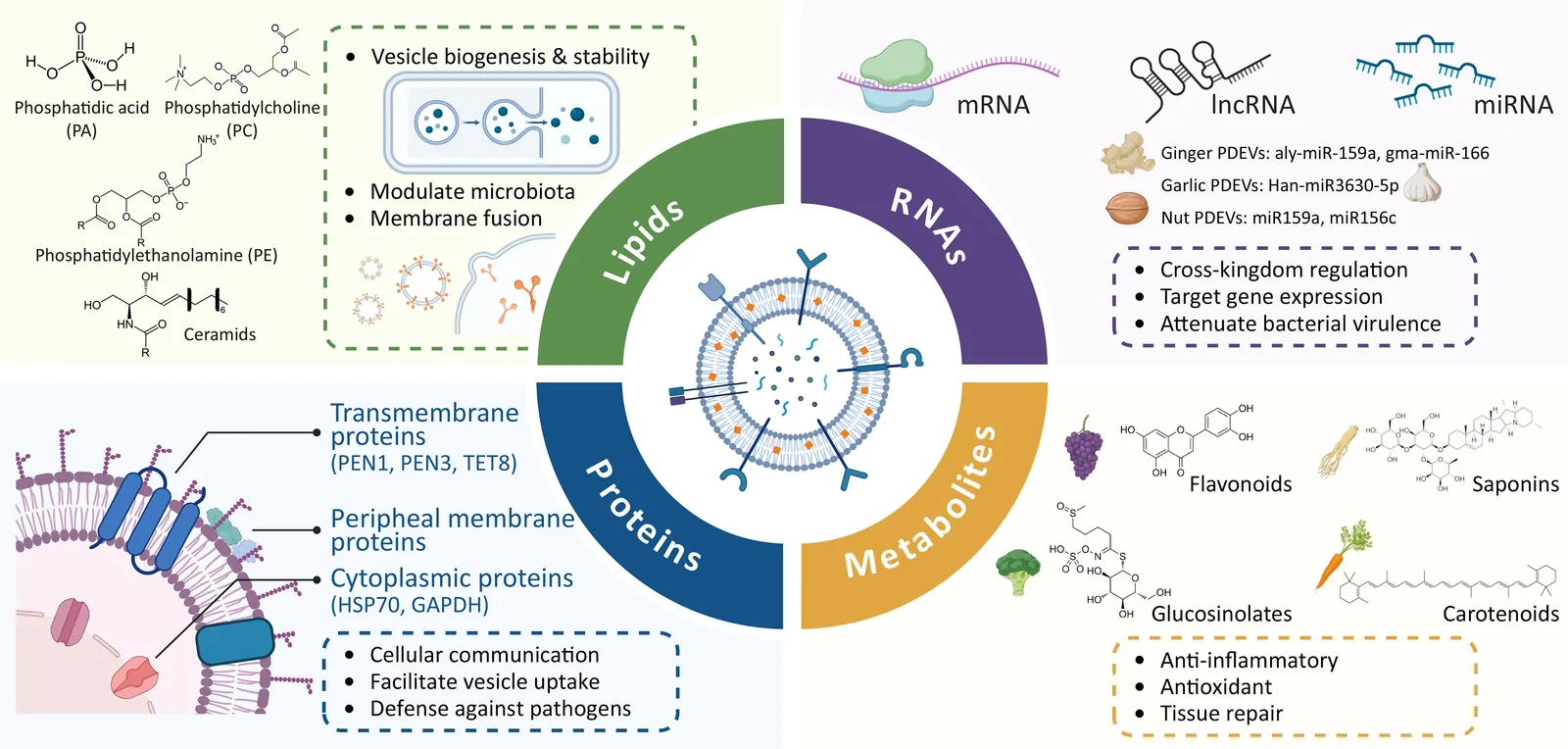
Molecular composition and functions of PDEVs. PDEVs contain lipids, RNAs, proteins, and metabolites that contribute to vesicle stability, cross-kingdom regulation, cellular communication, antimicrobial defence, anti-inflammatory and antioxidant effects, and tissue repair. Created with BioRender.com and subsequently modified and finalised using Adobe Illustrator. https://BioRender.com/4igus3g. PDEVs: Plant-derived extracellular vesicles.

### Lipids

Lipids are essential constituents of PDEVs, playing crucial roles in maintaining vesicle stability and facilitating diverse biological functions^[29]^. Lipid components commonly detected in PDEVs include phosphatidic acid (PA), phosphatidylethanolamine (PE), and phosphatidylcholine (PC), as well as ceramides and triglycerides^[29,48]^. Lipid profiles vary substantially among PDEVs derived from different botanical sources. For instance, PDEVs derived from aloe gel contain a high proportion of glucosylceramides (43.55%), followed by PA (18.12%), ceramides (14.98%), and PC (3.85%)^[49]^. PDEVs extracted from *Panax notoginseng* contain a substantial amount of ceramides (26.4%) and PA (21.9%). Additionally, these vesicles are composed primarily of unsaturated fatty acids (38.2%). Ginseng exosome-like nanoparticles consist primarily of phospholipids enriched in PC (28.8%), triglycerides (16.8%), and ceramides (12.9%).

Lipids are crucial for PDEV biogenesis and release. The absence of glycosyl-inositol-phospho-ceramides (GIPCs) in *Arabidopsis* leaf cells results in reduced PDEV secretion. Moreover, the exogenous application of GIPCs to the surface of *Arabidopsis* leaves promotes PDEV biogenesis and release^[50]^. PA promotes the aggregation of cytoplasmic proteins toward the membrane, potentially influencing vesicle formation. Additionally, unsaturated fatty acid moieties in PA, PE, and PC induce alterations in vesicle morphology, potentially enhancing membrane fusion dynamics^[51]^. Furthermore, variations in lipid composition influence PDEV uptake by specific gut microbiota. This suggests that specific lipid profiles could be leveraged for targeted therapeutic interventions^[52]^. Lipid composition also contributes to the intrinsic therapeutic efficacy of PDEVs. Specific lipid components play a pivotal role in membrane-mediated signalling, microbiota modulation, antioxidant and anti-inflammatory activity, and regulation of the tumour microenvironment^[41,53-56]^.

### Proteins

Major PDEV proteins include peripheral membrane proteins, transmembrane proteins, and intracellular proteins^[29]^. Three common protein families - HSP70, S-adenosyl-homocysteinase, and glyceraldehyde 3-phosphate dehydrogenase (GAPDH) - have been identified in PDEVs^[8]^. Transmembrane or GPI-anchored proteins, such as PEN1, PEN3, and TET8, as well as cytoplasmic proteins, including heat shock proteins and GAPDH, serve as promising candidate biomarkers. Membrane proteins not only mediate cellular communication and serve as recognition targets for PDEVs isolation but also facilitate vesicle uptake and internalisation by mammalian cells^[6,8]^. Aquaporins present on broccoli-derived PDEVs have been shown to maintain membrane stability^[57]^. Notably, cytosolic proteins in PDEVs are associated with cell wall remodelling and defence against invading pathogens^[58,59]^. Proteins contribute to several biological effects of PDEVs, including their immunomodulatory and antitumour activities; however, these effects may also involve lipids, RNAs and secondary metabolites^[56,60,61]^.

### RNAs

Diverse nucleic acids, including DNA, mRNA, microRNA (miRNA), and other non-coding RNAs, are encapsulated in PDEVs, with RNAs playing a prominent role in regulating biological functions^[53]^. These RNAs regulate gene expression and recipient cell function through cross-species interactions^[62,63]^. MiR-7972, found in exosome-like nanoparticles derived from fresh *Rehmanniae Radix* (a traditional Chinese medicinal herb), alleviates LPS-induced lung inflammation and ameliorates gut microbiota dysbiosis by targeting the GPR161-mediated Hedgehog pathway^[64]^. Ginger-derived nanovesicles enriched with miRNAs, such as aly-miR-159a and gma-miR-166, attenuate the virulence of *Porphyromonas gingivalis* (*P. gingivalis*) and reduce bacterial adhesion to gingival epithelial cells^[65]^. MiRNAs in exosome-like nanoparticles derived from *Houttuynia cordata* demonstrate potential antiviral activity against respiratory RNA viruses^[66]^. The 3′-ends of plant miRNAs are naturally modified by 2′-O-methylation, protecting them from degradation and uridylation, thereby enhancing their potential as therapeutic agents^[62]^. These findings underscore the profound impact of PDEVs and their associated miRNAs on cellular behaviour, inflammatory responses, and tissue repair. However, further research regarding the specific mechanisms of PDEV-derived miRNAs remains warranted.

### Metabolites

In addition to the aforementioned cargoes, PDEVs contain various metabolites crucial to their bioactivity, including carotenoids, flavonoids, saponins, and glucosinolates^[67,68]^. For instance, ginger-derived nanovesicles contain bioactive constituents, including 6-, 8-, and 10-gingerol and 6-shogaol, and exhibit anti-inflammatory and antioxidant effects in experimental models of inflammatory bowel disease and alcohol-induced liver injury^[69-71]^. Grapefruit-derived nanovesicles have been associated with the bioactive flavonoids naringin and naringenin, which may contribute to their pharmacological effects^[55,72]^. Broccoli-derived nanoparticles alleviate colitis by activating adenosine monophosphate-activated protein kinase (AMPK) in dendritic cells, a process mediated by sulforaphane^[73]^. In conclusion, various metabolites in PDEVs exhibit potential therapeutic functions; however, further elucidation of specific metabolic profiles and their molecular targets is required.

## BIOLOGICAL FUNCTIONS AND POTENTIAL THERAPEUTIC APPLICATIONS OF PDEVs IN DENTAL DISEASES

Before elaborating on the specific biological activities of PDEVs, it is informative to compare their translational attributes with those of mammalian cell-derived EVs, which have been far more thoroughly explored in dental and craniofacial regeneration. Mammalian cell-derived EVs, especially those originating from dental and mesenchymal stem cells, exhibit promising capacity for dental pulp, periodontal, and alveolar bone regeneration via the promotion of angiogenesis, tissue repair, and immunomodulation^[74,75]^. Nevertheless, the majority of supporting evidence remains restricted to pre-clinical observations. Relative to mammalian EVs, PDEVs feature renewable starting materials, comparatively low manufacturing costs, and superior scalability^[16,76]^. Nevertheless, mammalian EVs currently have better-characterised biogenesis, molecular markers, and tissue-specific mechanisms, whereas PDEVs are limited by heterogeneous isolation products, source-dependent cargo variation, insufficiently validated markers, and limited pharmacokinetic and clinical evidence^[16,76]^. Accordingly, PDEVs and mammalian dental-derived EVs should be considered complementary translational platforms. Against this translational backdrop, PDEVs exhibit three principal, interrelated biological activities - antimicrobial effects, anti-inflammatory and immunomodulatory actions, and tissue-regenerative promotion - that collectively underpin their therapeutic potential in dental disease management [Figure 3].

**Figure 3:**
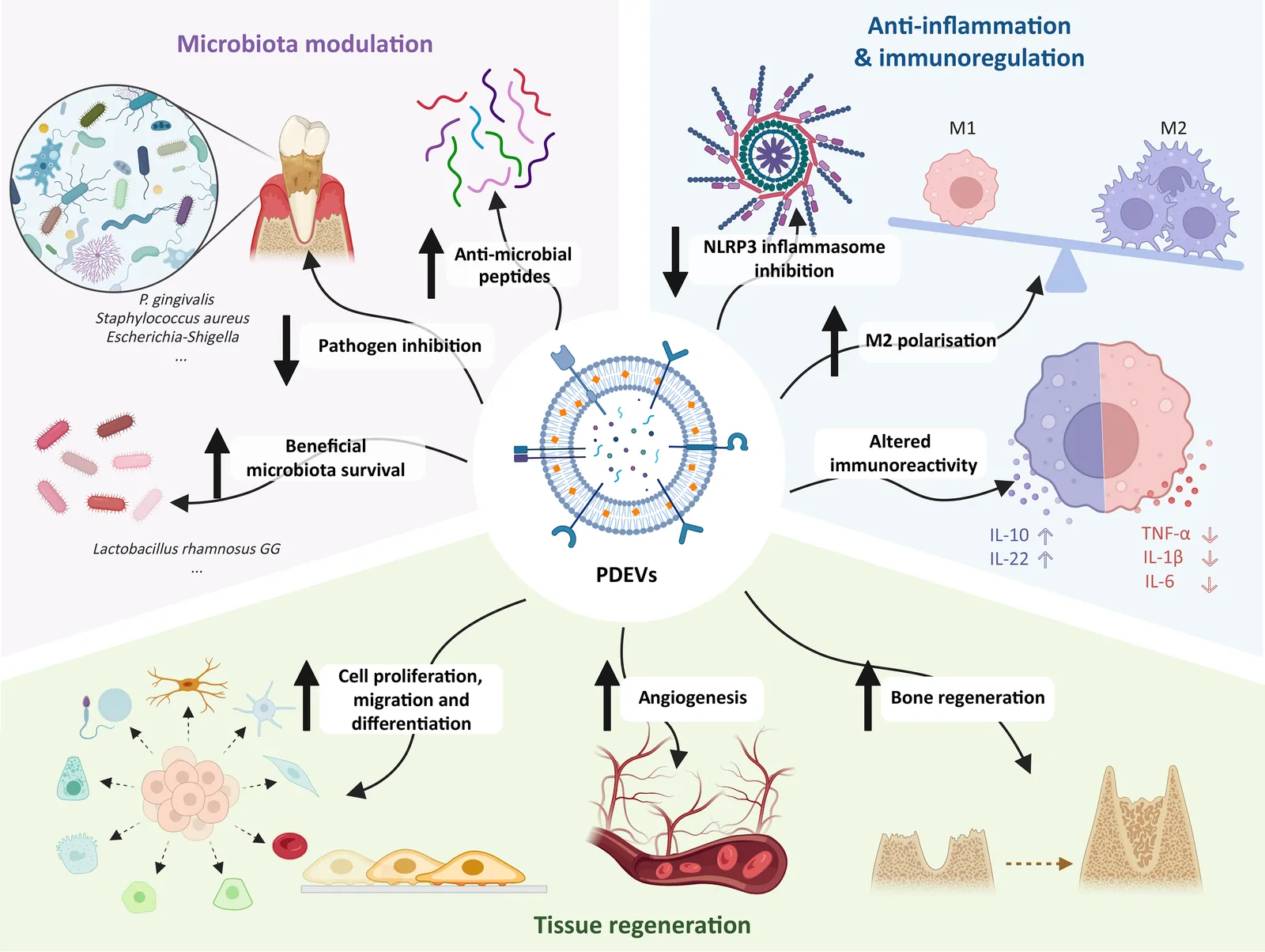
Biological functions of PDEVs. PDEVs modulate the microbiota and promote anti-inflammatory, immunoregulatory, and tissue-regenerative responses. Arrows indicate increases or decreases. Created with BioRender.com and subsequently modified and finalised using Adobe Illustrator. https://BioRender.com/y3bwt5a. IL: Interleukin; NLRP3: NOD-like receptor family pyrin domain-containing 3; PDEVs: plant-derived extracellular vesicles; *P. gingivalis*: *Porphyromonas gingivalis*; TNF-α: tumour necrosis factor-alpha.

### Modulation of microbiota

The oral cavity is colonized by a diverse array of microorganisms from birth. Collectively referred to as the oral microbiota, these microorganisms play a complex role in health, yet dysbiosis can precipitate various oral diseases. Species strongly associated with the initiation and progression of caries include *Streptococcus mutans*, *Lactobacillus* spp., *Actinomyces* spp., *Bifidobacterium* spp., and *Scardovia* spp.^[77]^. Pulpitis and apical periodontitis result from polymicrobial infections involving *streptococci*, *staphylococci*, and various anaerobic genera, such as *Fusobacterium*, *Porphyromonas*, *Prevotella*, and *Treponema*^[78]^. *Enterococcus faecalis* has been identified as the predominant pathogen associated with root canal treatment failure^[79]^. Periodontitis is closely linked to the "red complex" - comprising *P. gingivalis*, *Tannerella forsythia*, and *Treponema denticola* - which are regarded as the primary etiological agents^[80]^. A distinct bacterial profile has been characterized in peri-implantitis, featuring *P. gingivalis*, *Staphylococcus aureus* (*S. aureus*), *Staphylococcus anaerobius*, *Streptococcus intermedius*, *Streptococcus mitis*, *T. forsythia*, and *T. socranskii*^[81]^.

PDEVs demonstrate remarkable potential in combating pathogens and maintaining microbial homeostasis [Table 1]. For instance, ginger-derived nanovesicles are selectively endocytosed by the periodontal pathogen *P. gingivalis*. These vesicles attenuate bacterial virulence via specific lipids (notably PA 34:2) and miRNAs (such as aly-miR159a), thereby promoting microbial homeostasis^[65]^. Similarly, ginger-derived PDEVs alleviate DSS-induced colitis in murine models by targeting the gut bacterium *Lactobacillus rhamnosus* GG (LGG)^[52]^. Garlic-derived exosome-like nanovesicles exert antimicrobial activity against *S. aureus*, eliminating intracellular infection in macrophages and facilitating wound healing^[82]^. In a murine colitis model, pretreatment with garlic-derived exosome-like nanovesicles (GENs) favourably altered the gut microbiota profile, characterized by increased *Lachnospiraceae* abundance and decreased *Helicobacter* load^[83]^. Bee pollen-, honey- and royal jelly-derived nanovesicles substantially inhibit colony and biofilm formation in *S. aureus*^[84]^. Oral administration of turmeric-derived PDEVs reduced *Escherichia-Shigella* and *Helicobacter* levels in mice with ulcerative colitis^[85]^. Lemon-derived nanovesicles promote the survival of beneficial gut microbiota by downregulating the expression of methylation-specific restriction endonucleases (Msp1 and Msp3) in LGG^[86]^. Exosome-like nanoparticles (ELNs) were isolated from fresh *Rehmanniae Radix*, a well-known traditional Chinese herb used in the treatment of inflammatory and metabolic diseases. Rgl-miR-7972 (miR-7972) was identified as the primary component in these ELNs. MiR-7972 downregulated the expression of G protein-coupled receptor 161 (GPR161), thereby activating the Hedgehog pathway, and inhibited *Escherichia coli* biofilm formation by targeting the virulence gene stx2^[64]^. Exosome-like nanoparticles derived from edible mulberry bark exert species-specific growth-inhibitory effects on virulent *Listeria monocytogenes EGD* (*Lis-EGD*) by inducing the production of antimicrobial peptides, and protect against colitis^[61]^. Driven by their unique lipid composition, tomato-derived EVs exert a selective influence on gut bacterial growth, stimulating probiotic *Lactobacillus* species and inhibiting opportunistic pathogens such as *Clostridioides difficile* and *Fusobacterium nucleatum.* Phospholipid profiling of tomato-derived EVs showed that their antibacterial activity was mediated by specific lipids^[87]^. Dandelion-derived extracellular vesicle-like nanoparticles exert antivirulence activity by specifically binding to S. aureus exotoxins, thereby protecting host cells^[88]^. Tartary buckwheat-derived nanovesicles modulate the gut microbiota by promoting the growth of both *Escherichia coli* (*E. coli*) *and Lactobacillus rhamnosus* (*L. rhamnosus*), increasing overall microbial diversity. Target gene prediction revealed that several microRNAs may target functional genes involved in physiological processes related to *E. coli* and *L. rhamnosus*^[89]^. More recently, Tartary buckwheat-derived exosome-like nanovesicles were engineered as dual carriers for chlorogenic acid and selenium nanoparticles, improving cargo stability during gastrointestinal digestion and producing synergistic metabolic regulatory effects^[90]^. Treatment with *Pueraria lobata*-derived exosome-like nanovesicles reduced the abundance of pathogenic intestinal strains in osteoporotic SD rats, a modulation associated with the regulation of the gut-derived metabolite trimethylamine-N-oxide (TMAO)^[91]^. Orally administered *Portulaca oleracea L.*-derived exosome-like nanoparticles maintained gut microbial diversity, promoted the growth of *Lactobacillus reuteri*, and increased indole derivative levels. These effects activated the aryl hydrocarbon receptor (AhR) in conventional CD4+ T cells, which in turn downregulated Zbtb7b expression and drove the reprogramming of conventional CD4+ T cells into CD4+CD8+ double-positive T cells (DP CD4+CD8+ T cells)^[92]^. A recent comparative analysis further showed that fresh and dried *Portulaca oleracea* yield exosome-like nanoparticles with differences in particle characteristics and molecular composition, highlighting the importance of source processing for production standardisation^[93]^. Therefore, given their capacity to modulate oral and gut microbiota, PDEVs represent a potential therapeutic modality for microbiota-associated oral diseases. However, the exploration of PDEVs from diverse botanical sources and the optimization of delivery methods for specific dental indications require further investigation.

**Table 1:** Modulation of microbiota

**Plant source**	**Target(s)**	**Mechanism**	**Outcome**	**Disease/model**	**Ref.**
Ginger	*P. gingivalis*	Lipids, particularly PA (34:2), and miRNAs including aly-miR159a	Inhibited bacterial growth and virulence and reduced periodontal bone loss	Periodontitis	[65]
Ginger	*L. rhamnosus* GG	mdo-miR7267-3p-mediated targeting of the monooxygenase *ycnE*	Promoted LGG growth and enhanced beneficial microbial activity	DSS-induced colitis	[52]
Garlic	*S. aureus*	Vesicle internalisation and delivery of bioactive cargo	Suppressed intracellular infection and promoted wound healing	*S. aureus*-infected wound	[82]
Garlic	Lachnospiraceae and *Helicobacter*	Han-miR3630-5p-associated microbiota regulation	Increased Lachnospiraceae and decreased *Helicobacter* abundance	Colitis	[83]
Bee pollen, honey and royal jelly	*S. aureus*	Not determined	Inhibited bacterial colony and biofilm formation and promoted wound healing	Wound healing	[84]
Turmeric	*Bacteroides*, *Escherichia-Shigella*, *Helicobacter* and *Staphylococcus*	PI3K-AKT-associated regulation	Reduced the abundance of potentially pathogenic bacteria	Ulcerative colitis	[85]
Lemon	*L. rhamnosus* GG	Downregulation of Msp1 and Msp3	Enhanced bacterial stress resistance and survival	Gut microbial stress model	[86]
Rehmanniae Radix	*E. coli*	miR-7972-mediated targeting of the virulence gene *stx2*	Inhibited biofilm formation and alleviated microbiota dysbiosis	Acute lung injury	[64]
Mulberry bark	*L. monocytogenes* EGD	HSPA8-AhR-COPS8 signalling and induction of antimicrobial peptides	Inhibited bacterial growth and protected against colitis	Colitis	[61]
Tomato	*Lactobacillus*, *C. difficile* and *F. nucleatum*	Specific bioactive lipids	Promoted *Lactobacillus* growth while inhibiting *C. difficile* and *F. nucleatum*	Intestinal dysbiosis	[87]
Dandelion	*S. aureus* exotoxins	Direct binding and neutralisation of exotoxins	Reduced toxin-mediated cytotoxicity and promoted infected-wound healing	*S. aureus* exotoxin-infected wound	[88]
Tartary buckwheat	*E. coli* and *L. rhamnosus*	Putative regulation by vesicular miRNAs	Promoted bacterial growth and increased microbial diversity	*In vitro* gut microbiota model	[89]
*Pueraria lobata*	Pathogenic intestinal bacteria, including *Clostridium*	Regulation of microbiota-derived TMAO	Reduced pathogenic bacterial abundance and alleviated osteoporosis	Osteoporosis	[91]
*Portulaca oleracea* L.	*L. reuteri*	Microbiota-indole-AhR-Zbtb7b axis	Promoted *L. reuteri* growth, preserved microbial diversity and alleviated colitis	DSS-induced colitis	[92]

*C. difficile*: *Clostridioides difficile*; *E. coli*: *Escherichia coli*; *F. nucleatum*: *Fusobacterium nucleatum*; *L. monocytogenes EGD*: *Listeria monocytogenes EGD*; *L. rhamnosus GG*: *Lactobacillus rhamnosus GG*; *L. reuteri*: *Lactobacillus reuteri*; PA: phosphatidic acid; *P. gingivalis*: *Porphyromonas gingivalis*; *S. aureus*: *Staphylococcus aureus*; TMAO: trimethylamine-N-oxide.

### Anti-inflammatory effects and immunoregulation

Inflammation is the immune system’s physiological response to noxious stimuli, including pathogens, damaged cells, or irritants. Although essential for pathogen defence and tissue repair, severe or chronic inflammation often results in irreversible tissue damage. Pulpitis, characterised by inflammation of the dental pulp, typically arises from untreated caries, trauma, or restorative procedures^[94]^. Apical periodontitis results from pulpal infection and is characterised by the destruction and resorption of periapical bone^[95]^. Periodontitis is a chronic inflammatory disease affecting the supporting periodontal structures, including the gingiva, periodontal ligament, and alveolar bone. This condition can lead to tooth loss and exacerbate systemic diseases^[96]^. Peri-implantitis is a biofilm-induced inflammatory condition. It affects the soft and hard tissues surrounding dental implants, resulting in bone loss and potential implant failure^[97]^. Although therapeutic modalities for these oral diseases vary, a common objective is to restore the immune microenvironment and reduce inflammation.

Extensive literature has documented the anti-inflammatory properties of PDEVs and their therapeutic efficacy in inflammatory diseases [Table 2]. Ginger exosome-like nanoparticles demonstrate therapeutic potential for periodontitis by alleviating oxidative stress and inflammation of periodontal ligament fibroblasts (PDLFs) via Nuclear factor kappa B (NF-κB) pathway inhibition^[98]^. Additionally, Ginger exosome-like nanoparticles inhibit intestinal inflammation by modulating macrophage polarisation. Mechanistically, osa-miR164d directly targets the 3′-UTR of TGF-β-activated kinase 1-binding protein 1 (TAB1) and regulates macrophage polarisation by downregulating NF-κB expression^[99]^. Similarly, in colitis models, oral administration of ginger-derived PDEVs exerts anti-inflammatory effects. This is achieved by downregulating pro-inflammatory cytokines, such as tumour necrosis factor alpha (TNF-α), interleukin-6 (IL-6), and IL-1β, while simultaneously upregulating anti-inflammatory cytokines, including IL-10 and IL-22^[69]^. EVs derived from *Pueraria lobata* (Gegen) - a dual-purpose edible and medicinal herb - induce a transition from M1 to M2 macrophage phenotypes and substantially decrease proinflammatory factor expression^[100]^. Goldenberry-derived exosome-like nanoparticles (GDENs) exhibit anti-inflammatory properties by reducing M1 macrophage activity and promoting M2 polarisation^[101]^. Folic acid-conjugated ginger-derived extracellular vesicles (FA-GDEVs), produced by conjugating GDEVs with folic acid, promote macrophage polarisation toward a reparative M2 phenotype *in vitro* via the PI3K-AKT pathway. Further *in vivo* evidence indicates that FA-GDEVs accumulate in arthritic joints and markedly alleviate the symptoms of rheumatoid arthritis^[102]^. Broccoli-derived nanoparticles exert protective effects against DSS-induced colitis by activating AMPK in dendritic cells, which suppresses their activation and promotes a tolerogenic phenotype^[73]^. Grapefruit-derived PDEVs (GDNs) alleviate DSS-induced colitis without detectable toxicity. Specifically, intestinal macrophages internalise GDNs, resulting in the upregulation of heme oxygenase-1 (HO-1) expression and the concomitant reduction of IL-1β and TNF-α production^[55]^. PDEVs derived from cabbage and red cabbage protect RAW264.7 cells from LPS-induced inflammation by attenuating pro-inflammatory cytokines (IL-6 and IL-1β) and Cyclooxygenase-2 (COX-2) levels^[103]^. Garlic chives-derived PDEVs suppress NOD-like receptor family pyrin domain-containing 3 (NLRP3) inflammasome activation and chronic inflammation^[104]^. Blueberry-derived PDEVs protect EA.hy926 cells from TNF-α-induced cytotoxicity and oxidative stress^[105]^. Exosome-like nanovesicles derived from strawberry juice (*Fragaria* × *ananassa*, cv. Romina) protect human mesenchymal stromal cells (MSCs) from oxidative stress, a mechanism potentially attributed to their vitamin C content^[106]^. Tea leaf-derived nanotherapeutics exert protective effects against inflammatory bowel disease and colitis-associated colon cancer by downregulating pro-inflammatory responses, alleviating oxidative stress, and preserving intestinal microbial homeostasis^[107]^. Exosome-like nut nanovesicles transport microRNAs, such as miR159a and miR156c, which downregulate Tnfrsf1a expression and suppress TNF-α signalling, thereby attenuating inflammatory responses^[108]^.

**Table 2:** Anti-inflammatory effects and immunoregulation

**Plant source**	**Target(s)**	**Mechanism**	**Outcome**	**Disease/model**	**Ref.**
Ginger	PDLFs	Inhibition of NF-κB signalling	Reduced oxidative stress and inflammatory responses	Periodontitis	[98]
Ginger	Macrophages	osa-miR164d-mediated targeting of TAB1 and downregulation of NF-κB	Suppressed M1 polarisation and promoted the anti-inflammatory macrophage phenotype	Intestinal inflammation	[99]
Ginger	Intestinal epithelial cells and macrophages	Downregulation of TNF-α, IL-6 and IL-1β and upregulation of IL-10 and IL-22	Reduced intestinal inflammation and promoted mucosal repair	DSS-induced colitis	[69]
*Pueraria lobata*	Macrophages	M1-to-M2 macrophage polarisation	Reduced M1-associated inflammatory mediators and increased M2-associated markers	*In vitro* macrophage-polarisation model	[100]
Goldenberry	Macrophages	Suppression of M1 activation and promotion of M2 polarisation	Reduced pro-inflammatory macrophage responses	Macrophage inflammation model	[101]
Folic acid-modified ginger EVs	Macrophages	PI3K-AKT-mediated M2 polarisation	Promoted reparative M2 polarisation and alleviated joint inflammation	Rheumatoid arthritis	[102]
Broccoli	Dendritic cells	AMPK activation and induction of tolerogenic dendritic cells	Suppressed dendritic-cell activation and alleviated intestinal inflammation	DSS-induced colitis	[73]
Grapefruit	Macrophages	HO-1 upregulation and inhibition of IL-1β and TNF-α production	Reduced macrophage-mediated inflammation and attenuated colitis	DSS-induced colitis	[55]
Cabbage and red cabbage	RAW264.7 macrophages	Suppression of IL-6, IL-1β and COX-2	Attenuated LPS-induced inflammatory responses	LPS-induced inflammation	[103]
Garlic chives	Macrophages	Inhibition of NLRP3 inflammasome-associated pathways	Reduced inflammasome activation and chronic inflammation	Obesity-associated inflammation	[104]
Blueberry	EA.hy926 endothelial cells	Attenuation of TNF-α-induced oxidative stress	Reduced cytotoxicity and oxidative damage	Vascular inflammation model	[105]
Strawberry	MSCs	Antioxidant activity	Protected MSCs against oxidative stress	Oxidative stress model	[106]
Tea leaf	Macrophages and intestinal cells	Suppression of pro-inflammatory cytokines and enhancement of IL-10	Reduced inflammation and oxidative stress and preserved microbial homeostasis	Inflammatory bowel disease and colitis-associated cancer	[107]
Nut	Adipocytes	Delivery of miR159a and miR156c and suppression of TNF-α signalling	Reduced inflammatory signalling and improved adipocyte metabolism	Inflammation-associated metabolic disease	[108]

AMPK: AMP-activated protein kinase; COX-2: cyclooxygenase-2; HO-1: heme oxygenase-1; IL: interleukin; MSCs: mesenchymal stromal cells; NF-κB: nuclear factor kappa B; NLRP3: NOD-like receptor family pyrin domain-containing 3; PDLFs: periodontal ligament fibroblasts; TAB1: TGF-β-activated kinase 1-binding protein 1; TNF-α: tumour necrosis factor alpha.

Collectively, despite their diverse botanical origins and source-specific cargo profiles, PDEVs converge on several shared anti-inflammatory and immunomodulatory mechanisms. Ginger- and garlic-derived vesicles suppress NF-κB signalling through mechanisms involving TAB1 or the TLR4/MyD88 axis^[83,98,99]^. Macrophage reprogramming represents another conserved mode of action: vesicles derived from ginger, *Pueraria lobata*, goldenberry, and folic acid-modified ginger suppress the pro-inflammatory M1 phenotype and/or drive reparative M2 polarisation through pathways such as NF-κB and PI3K-AKT^[99-102]^. Additional PDEVs exert effects through complementary pathways: garlic chive-derived vesicles inhibit the NLRP3 inflammasome; grapefruit-derived vesicles induce HO-1 expression; broccoli-derived nanoparticles elicit AMPK-dependent dendritic-cell tolerance; and vesicles from blueberry, strawberry, and tea leaves mitigate oxidative stress^[55,73,104-107]^. Accordingly, macrophage-targeted immunomodulation is a prominent feature of vesicles from ginger, *Pueraria lobata*, and goldenberry, whereas preparations from other plant sources display more marked inflammasome-regulatory, tolerogenic, or antioxidant activities. While these functional categories partially overlap, direct head-to-head comparisons are still scarce. Their consistent actions on inflammatory signalling cascades, immune-cell reprogramming, and redox homeostasis warrant further exploration of PDEVs for managing inflammatory dental diseases.

### Regenerative capacity

Regenerative endodontics aims to restore the vitality and function of the pulp-dentine complex. Regenerating periodontal tissues - specifically cementum, periodontal ligament fibres, and alveolar bone - remains a substantial challenge in periodontal therapy. Alveolar bone defects primarily result from periodontal disease, trauma, tumour resection, genetic factors, or systemic pathologies, thereby complicating restorative interventions such as dental implants and removable dentures. Biological processes, including pulp regeneration, osteogenesis, angiogenesis, and tissue remodelling, critically influence dental tissue regeneration outcomes.

PDEVs demonstrate remarkable regenerative capabilities, promoting cell proliferation, inducing cellular differentiation, and stimulating angiogenesis [Table 3]. *Aloe saponaria-*derived nanovesicles triple the proliferation capacity of human dermal fibroblasts (HDFs)^[109]^. EVs isolated from *Opuntia ficus-indica* fruit accelerate wound healing by promoting HDF migration^[110]^. Exosome-like vesicles derived from *Lithospermum erythrorhizon* callus promote key wound-healing functions in normal HDFs, including viability, proliferation, wound closure, and collagen I synthesis^[111]^. Wheat exosomes promote the proliferation and migration of endothelial, epithelial, and dermal fibroblasts in a concentration-dependent manner, with optimal efficacy observed at 200 µg/mL^[112]^. Bitter melon-derived nanovesicles also stimulate cell proliferation dose-dependently and confer cytoprotection against radiation-induced damage^[113]^. Grape-derived exosome-like nanoparticles promote intestinal epithelial regeneration during both homeostasis and injury repair^[114]^. Cabex and Rabex not only promote mammalian cell proliferation but also suppress inflammation in immune cells^[103]^. Notably, yam-derived nanovesicles promote osteogenic differentiation and mineralisation by activating the BMP-2/p-P38/Runx2 signalling pathway^[115]^. Similarly, apple-derived nanovesicles enhance osteogenic gene and protein expression in MC3T3-E1 osteoblasts by modulating the BMP-2/Smad-1 pathway^[116]^. Plum-derived exosome-like nanovesicles promote the proliferation, differentiation, and mineralisation of both MC3T3-E1 osteoblasts and primary murine bone marrow-derived osteoblasts^[117]^. *Pueraria lobata*-derived exosome-like nanovesicles enhance osteogenic differentiation and mineralisation in human bone marrow mesenchymal stem cells (BMSCs) *in vitro* and in ovariectomized osteoporotic rats^[91]^. *Rhizoma Drynariae*-derived nanovesicles show therapeutic promise for postmenopausal osteoporosis by promoting BMSCs osteogenic differentiation via ERα targeting^[118]^. Ginseng-derived nanovesicles promote neural differentiation in BMSCs by delivering miRNAs targeting neural-related signalling pathways^[119]^. *Beta vulgaris* and its exosomes demonstrate multifunctional capacities: promoting angiogenesis by facilitating endothelial vascular network formation and exerting anti-aging and anti-scarring effects on fibroblasts^[120]^. These findings underscore the therapeutic potential of PDEVs in tissue regeneration by inducing cell proliferation and differentiation. Regenerating oral tissues - ranging from hard tissues (enamel, dentin, bone) to soft tissues (dental pulp, periodontal ligament) - is a critical objective in dentistry, for which PDEVs offer promising broad-spectrum potential.

**Table 3:** Regenerative capacity

**Plant source**	**Target(s)**	**Mechanism/outcome**	**Disease/model**	**Ref.**
*Aloe saponaria*	HDFs	Promoted cell proliferation, migration and angiogenesis	Wound healing	[109]
*Opuntia ficus-indica* fruit	HDFs	Promoted fibroblast migration and accelerated wound closure	Wound healing	[110]
*Lithospermum erythrorhizon* callus	HDFs	Enhanced cell viability, proliferation, wound closure and collagen I synthesis	Wound healing	[111]
Wheat	Endothelial, epithelial and dermal fibroblast cells	Promoted cell proliferation and migration	Wound healing	[112]
Bitter melon	H9C2 cardiomyocytes	Stimulated proliferation and reduced apoptosis and radiation-induced damage	Radiation-induced cardiac injury	[113]
Grape	Intestinal stem cells	Promoted intestinal stem-cell proliferation and epithelial regeneration	DSS-induced colitis	[114]
Cabbage and red cabbage (Cabex and Rabex)	HaCaT and RAW264.7 cells	Promoted cell proliferation and attenuated inflammatory responses	Cell proliferation and inflammation models	[103]
Yam	Osteoblasts	Activated BMP-2/p38/Runx2 signalling and promoted differentiation and mineralisation	Osteoporosis	[115]
Apple	MC3T3-E1 osteoblasts	Activated BMP-2/Smad1 signalling and enhanced osteogenic differentiation	Osteogenesis model	[116]
Plum	MC3T3-E1 and primary bone marrow-derived osteoblasts	Promoted osteoblast proliferation, differentiation and mineralisation and reduced osteoclast activation	Osteogenesis and osteoporosis models	[117]
*Pueraria lobata*	BMSCs	Enhanced autophagy, osteogenic differentiation and mineralisation	Osteoporosis	[91]
*Rhizoma Drynariae*	BMSCs	Targeted ERα signalling and promoted osteogenic differentiation	Postmenopausal osteoporosis	[118]
Ginseng	BMSCs	Delivered regulatory miRNAs and promoted neural differentiation	Neural regeneration	[119]
*Beta vulgaris*	Endothelial cells and fibroblasts	Promoted angiogenesis and exerted anti-ageing and anti-scarring effects	Angiogenesis and tissue-repair models	[120]

BMSCs: Bone marrow mesenchymal stem cells; HDFs: human dermal fibroblasts.

## POSSIBLE APPLICATIONS OF PDEVs IN DIFFERENT DENTAL DISEASES

### Dental caries

Dental caries is a biofilm-mediated, sugar-driven disease. In its incipient stages, dental caries can be arrested or reversed via fluoride treatments - such as fluoride varnishes, mouthwashes, or dentifrices - which promote enamel remineralisation. However, once cavitation occurs, the carious tissue must be excised and the structure restored using restorative materials, typically composite resin or dental amalgam^[121]^. In the management of deep carious lesions, calcium hydroxide has traditionally served as the liner of choice for maintaining pulp vitality. This material protects the pulp-dentin complex via multiple mechanisms: reducing bacterial load, promoting dentin remineralisation, stimulating reparative dentin formation, and providing a barrier against cytotoxic agents. However, its antibacterial efficacy remains limited^[122,123]^.

Direct evidence demonstrating the inhibitory effects of isolated PDEVs on *Streptococcus mutans*, cariogenic biofilms, or dental caries development is still lacking. As a relevant proof-of-concept finding, honey-derived EVs have been shown to suppress *S. mutans* growth and biofilm formation, exhibiting stronger antibacterial activity against *S. mutans* than the oral commensal strain *Streptococcus sanguinis*^[124]^. However, honey-derived EVs originate from a bee product and should not be classified as PDEVs. Available plant-derived evidence remains indirect: ginger-, tomato-, and garlic-derived nanovesicles inhibit or attenuate the virulence of *P. gingivalis*, *Fusobacterium nucleatum*, and *S. aureus*, respectively^[65,82,87]^. Future investigations are therefore warranted to systematically evaluate the impacts of PDEVs on *S. mutans* virulence, glucosyltransferase activity, extracellular polysaccharide synthesis, acid production, cariogenic biofilm maturation, and subsequent enamel demineralisation^[125]^. PDEVs may potentially be incorporated into mouthrinses, dentifrices, varnishes, or restorative materials, although their oral retention, stability, safety, and effects on microbial homeostasis require further investigation.

### Infected pulp diseases

Following pulp exposure, vital pulp therapy (VPT) - including pulp capping, partial pulpotomy, or full pulpotomy - may be indicated depending on the pulpal diagnosis, restorability of the tooth, and ability to control pulpal bleeding. If adequate haemostasis cannot be achieved after removal of the coronal pulp, pulpectomy and subsequent root canal therapy are required. Ideal VPT materials must be antimicrobial, radiopaque, biocompatible (inducing minimal irritation), and dentinogenic (promoting tertiary dentin formation), while also providing an effective seal against microleakage, ease of handling, and resistance to displacement. Although hydraulic calcium silicate cements, such as mineral trioxide aggregate (MTA) and Biodentine, demonstrate superior histological responses and outperform the historical gold standard, calcium hydroxide, they lack the optimal property profile required for comprehensive VPT^[123,126]^. Root canal therapy represents the standard of care for managing infected dental pulp. This intervention involves the meticulous extirpation of infected pulp tissue, followed by the thorough chemo-mechanical preparation and disinfection of the root canal system. The canal system is subsequently obturated to prevent reinfection. Persistent intraradicular infection is a major cause of endodontic failure, and *Enterococcus faecalis* is one of the microorganisms most frequently associated with persistent or secondary endodontic infections^[127]^. Although sodium hypochlorite remains the most widely used root canal irrigant, its limitations include concentration-dependent cytotoxicity, an unpleasant taste and potential adverse effects on the structural and mechanical properties of root dentine, particularly at high concentrations or after prolonged exposure^[128,129]^. In endodontic therapy, root canal sealers are used together with gutta-percha points, serving to fill the gaps between the filling material and the dentinal wall to achieve a hermetic seal of the canal system. An ideal root canal sealer should possess excellent sealing ability, biocompatibility, antimicrobial properties, radiopacity, and optimal handling characteristics^[130]^. Root canal sealers play a critical role in sealing endodontically treated teeth, yet they present notable drawbacks. Their primary disadvantage is the extreme difficulty of removal during root canal retreatment. Other concerns include potential cellular toxicity, inflammatory potential, and moisture sensitivity. Over time, they may shrink and compromise the seal, or expand excessively and cause root fracture^[131]^. Recent research has focused on improving the biological and physical properties of root canal sealers by incorporating bioactive materials that may contribute to periapical tissue healing and regeneration. However, direct evidence supporting PDEVs for pulpitis, apical periodontitis, or root canal disinfection is currently unavailable. Their reported antimicrobial, anti-inflammatory, and osteogenic activities provide a mechanistic rationale for incorporation into pulp-capping materials, intracanal medicaments, irrigants, or sealers; nevertheless, these applications remain conceptual. Future investigations should assess their activity against endodontic pathogens, particularly *E. faecalis*, as well as their penetration into dentinal tubules, compatibility with dental pulp cells, and effects on reparative dentin formation and periapical healing.

### Periodontitis and peri-implantitis

Scaling and root planing is the cornerstone of nonsurgical periodontal therapy, whereas peri-implantitis is initially managed through mechanical debridement and implant-surface decontamination. However, the efficacy of scaling and root planing (SRP) is constrained by limited access to deep periodontal pockets and complex root anatomies, the risk of postoperative hypersensitivity, and an inability to restore alveolar bone lost to severe periodontitis^[132]^. Although local and systemic pharmacotherapies offer adjunctive benefits, their clinical utility is circumscribed by risks such as antimicrobial resistance, hypersensitivity reactions, and superinfection^[133]^. Regenerative surgical interventions employ diverse materials, ranging from autogenous bone grafts to alloplastic substitutes; however, few have demonstrated the capacity for true periodontal regeneration^[134]^. Direct pre-clinical evidence supports PDEV-based intervention for periodontitis. Ginger-derived exosome-like nanoparticles selectively inhibited the growth and virulence of *P. gingivalis* and reduced periodontal bone loss in mice^[65]^. Subsequent studies showed that ginger-derived vesicles suppressed inflammatory and oxidative responses in PDLFs and attenuated periodontitis-associated tissue damage^[98]^. More recently, celecoxib-loaded *Allium cepa*-derived EVs inhibited osteoclast differentiation and reduced alveolar bone loss in an experimental periodontitis model^[135]^. These findings support PDEVs as potential antimicrobial, immunomodulatory, and drug-delivery platforms for periodontitis. However, direct evidence in peri-implantitis remains lacking, and efficacy should not be assumed because implant-associated biofilms and peri-implant tissues differ from their periodontal counterparts. PDEVs may serve as antimicrobial, immunomodulatory, and regenerative agents for periodontitis and peri-implantitis. However, further studies using standardised disease-specific models are required to establish their efficacy, safety, and optimal local delivery strategies.

### PDEVs as drug-delivery carriers for dental applications

Beyond their intrinsic biological activities, PDEVs can be engineered to serve as natural nanocarriers for exogenous therapeutic cargo. Passive incubation typically maintains vesicle integrity yet often yields relatively low loading efficiency. By contrast, sonication, electroporation, freeze-thaw cycling and extrusion improve cargo loading capacity, but these manipulations may also alter vesicle architecture or trigger particle aggregation^[136]^. Hydrogels may further enhance local retention and controlled release within the oral cavity. Direct dental pre-clinical evidence has recently emerged. Oridonin-loaded nanovesicles derived from *Rabdosia rubescens* were embedded within a hydrogel, alleviating NLRP3-driven inflammation and accelerating wound healing in chemoradiotherapy-induced oral mucositis^[137]^. As described in Section “Periodontitis and peri-implantitis”, celecoxib-loaded *Allium cepa*-derived EVs provide direct proof of concept for locally delivered, drug-loaded PDEVs in periodontitis^[135]^. These findings support engineered PDEVs as local dental delivery platforms, although their loading efficiency, cargo stability, oral retention, release kinetics, batch consistency, and long-term safety require further evaluation. These potential applications of PDEVs in dental disease management are summarised in Figure 4.

**Figure 4:**
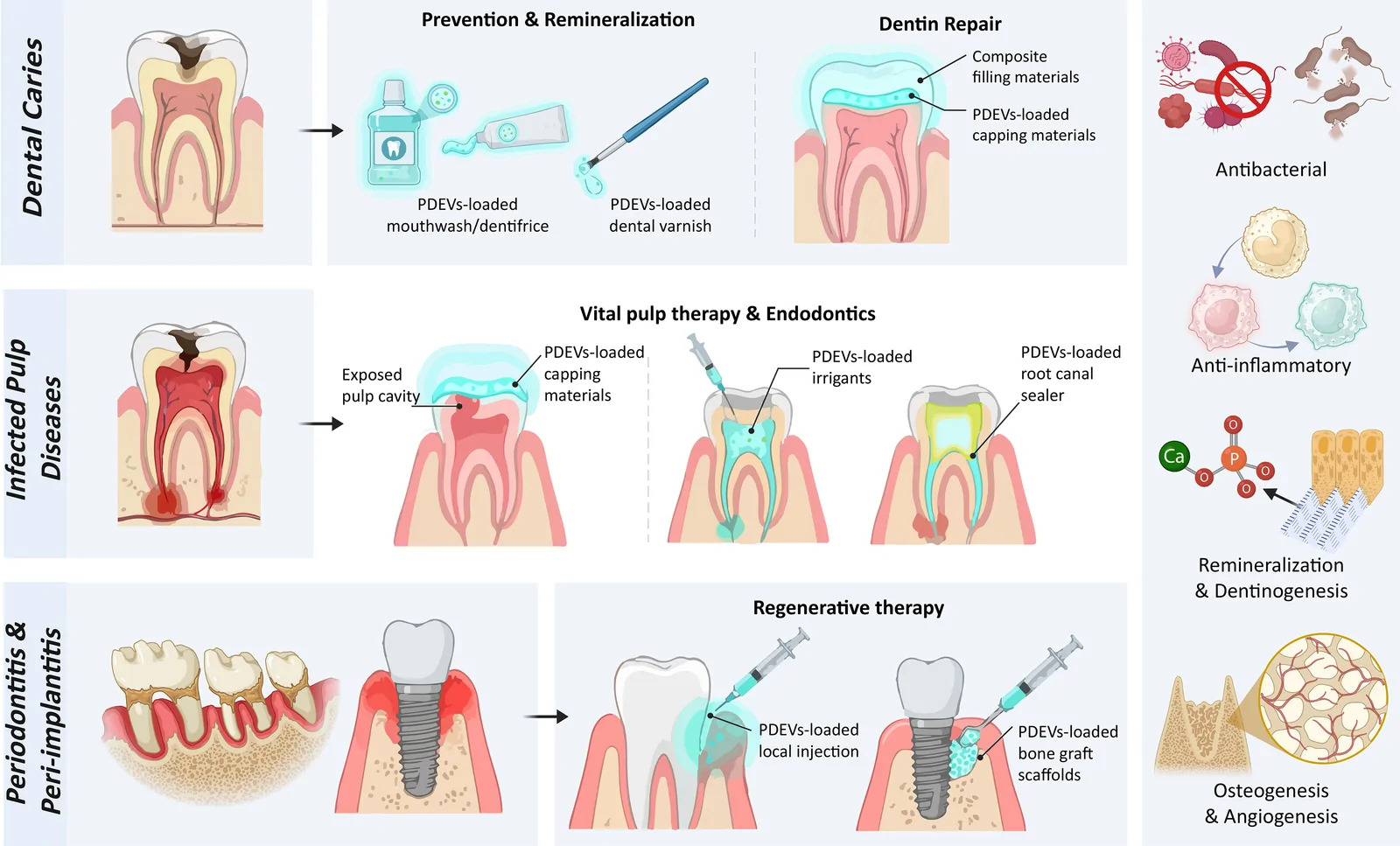
Potential applications of PDEVs in dental diseases. PDEVs may support the management of dental caries, pulp diseases, periodontitis, and peri-implantitis through antimicrobial effects, anti-inflammatory and immunomodulatory actions, and tissue-regenerative promotion. Created with BioRender.com and subsequently modified and finalised using Adobe Illustrator. https://BioRender.com/r41dref. PDEVs: Plant-derived extracellular vesicles.

## LIMITATIONS AND FUTURE CLINICAL DIRECTIONS

Despite their promising biological activities, several challenges must be addressed before PDEVs can be translated into dental practice. First, current evidence is derived predominantly from *in vitro* and animal studies, with direct evidence in dental disease-specific models remaining limited. Standardised comparisons of PDEVs from different plant sources should therefore be performed in models of dental caries, pulpitis, apical periodontitis, periodontitis, and peri-implantitis. Promising candidates should subsequently be evaluated in appropriately designed early-phase clinical trials to determine their safety, dosage, efficacy, and long-term outcomes.

Second, variations in plant species, cultivation conditions, tissue processing, isolation methods, and storage can substantially affect vesicle yield, composition, and biological activity. Future studies should establish critical quality attributes and standardised reporting criteria, including particle number, particle-to-protein ratio, size distribution, zeta potential, cargo composition, sterility, stability, and biological potency. Defining consistent dose units and batch-release criteria will be essential for reproducible and Good Manufacturing Practice-compliant production.

Third, efficient local delivery remains challenging because salivary flow, swallowing, oral movements, enzymatic degradation, mucosal barriers, and mature biofilms can reduce vesicle retention and therapeutic availability^[138,139]^. Mucoadhesive hydrogels, films, patches, and *in situ*-gelling formulations may protect PDEVs and prolong their residence time^[140]^. Surface engineering and optimisation of vesicle size and charge may further improve penetration into oral mucosa, periodontal pockets, dentinal tubules, or biofilms. A PDEV-loaded hydrogel has provided preliminary proof of concept that biomaterial-based delivery may prolong local vesicle retention and release in the oral cavity^[137]^.

Finally, PDEV selection should match the intended dental indication rather than assuming one plant source is universally optimal. Ginger-derived PDEVs may be prioritised for periodontal antimicrobial and anti-inflammatory applications, whereas osteogenic vesicles from yam, apple, or plum may be more suitable for bone-regenerative strategies. Drug-loaded *Allium cepa*- and *Rabdosia rubescens*-derived vesicle formulations provide early models for periodontal and oral mucosal delivery, respectively^[65,98,115-117,135,137]^. Future head-to-head studies should compare candidate sources using standardised measures of potency, safety, scalability, cargo preservation, and local retention before clinical development.

Regulatory classification, biosafety, and quality control also require clarification. Potential risks associated with plant allergens, pesticides, environmental contaminants, residual extraction reagents, unintended plant-derived bioactive cargo, and long-term immunological effects should be systematically assessed. Establishing regulatory guidance, traceable raw-material standards, and Good Manufacturing Practice-compliant production will be necessary for clinical translation.

### Limitations of this review

Several limitations should be considered when interpreting this review. First, as a narrative rather than a systematic review, relevant studies may have been missed, and publication or selection bias cannot be excluded. Second, much of the mechanistic evidence was obtained from non-dental disease models or *in vitro* and animal studies. Extrapolating these findings to oral tissues, the oral microbiome, and human dental diseases is therefore inferential, and some proposed dental applications remain conceptual. Third, substantial heterogeneity in PDEV isolation, characterisation, dose reporting, and experimental models limits direct comparisons of yield, purity, and bioactivity across plant sources. Consequently, the conclusions should be regarded as preliminary until validated using standardised protocols, dental disease-specific models, and well-designed clinical studies.

## CONCLUSION AND FUTURE PERSPECTIVES

Despite these limitations, PDEVs have garnered considerable attention owing to their abundance, favourable safety profile, and broad therapeutic potential, although research in this field remains at an early stage. The management of dental caries, pulpitis, apical periodontitis, periodontitis, and peri-implantitis requires microbiome modulation, inflammation control, immunoregulation, and tissue regeneration, all of which represent potential therapeutic targets for PDEVs. Future research should identify optimal plant sources, establish standardised production and delivery protocols, and evaluate efficacy and safety in dental disease-specific models and clinical studies.

## DECLARATIONS

### AI and AI-assisted tools statement

During the preparation of this manuscript, the AI tool ChatGPT (GPT-5.6 Sol, released 2026-07-09) was used solely for English-language editing and polishing. The tool did not influence the study design, data collection, analysis, interpretation, or the scientific content of the work. All authors take full responsibility for the accuracy, integrity, and final content of the manuscript.

### Copyright

© The Author(s) 2026.
